# Deep learning assessment of left ventricular hypertrophy based on electrocardiogram

**DOI:** 10.3389/fcvm.2022.952089

**Published:** 2022-08-11

**Authors:** Xiaoli Zhao, Guifang Huang, Lin Wu, Min Wang, Xuemin He, Jyun-Rong Wang, Bin Zhou, Yong Liu, Yesheng Lin, Dinghui Liu, Xianguan Yu, Suzhen Liang, Borui Tian, Linxiao Liu, Yanming Chen, Shuhong Qiu, Xujing Xie, Lanqing Han, Xiaoxian Qian

**Affiliations:** ^1^Department of Cardiology, The Third Affiliated Hospital of Sun Yat-sen University, Guangzhou, China; ^2^China Unicom (Guangdong) Industrial Internet Ltd., Guangzhou, China; ^3^Department of Endocrine and Metabolic Diseases, The Third Affiliated Hospital, Sun Yat-sen University, Guangzhou, China; ^4^LCFC (Hefei) Electronics Technology Co., Ltd., Hefei, China; ^5^Hefei LCFC Information Technology Co., Ltd., Hefei, China; ^6^Center for Artificial Intelligence, Research Institute of Tsinghua, Pearl River Delta, Guangzhou, China

**Keywords:** left ventricular hypertrophy, electrocardiogram, echocardiography, deep learning model, convolutional neural network-long short-term memory

## Abstract

**Background:**

Current electrocardiogram (ECG) criteria of left ventricular hypertrophy (LVH) have low sensitivity. Deep learning (DL) techniques have been widely used to detect cardiac diseases due to its ability of automatic feature extraction of ECG. However, DL was rarely applied in LVH diagnosis. Our study aimed to construct a DL model for rapid and effective detection of LVH using 12-lead ECG.

**Methods:**

We built a DL model based on convolutional neural network-long short-term memory (CNN-LSTM) to detect LVH using 12-lead ECG. The echocardiogram and ECG of 1,863 patients obtained within 1 week after hospital admission were analyzed. Patients were evenly allocated into 3 sets at 3:1:1 ratio: the training set (*n* = 1,120), the validation set (*n* = 371) and the test set 1 (*n* = 372). In addition, we recruited 453 hospitalized patients into the internal test set 2. Different DL model of each subgroup was developed according to gender and relative wall thickness (RWT).

**Results:**

The LVH was predicted by the CNN-LSTM model with an area under the curve (AUC) of 0.62 (sensitivity 68%, specificity 57%) in the test set 1, which outperformed Cornell voltage criteria (AUC: 0.57, sensitivity 48%, specificity 72%) and Sokolow-Lyon voltage (AUC: 0.51, sensitivity 14%, specificity 96%). In the internal test set 2, the CNN-LSTM model had a stable performance in predicting LVH with an AUC of 0.59 (sensitivity 65%, specificity 57%). In the subgroup analysis, the CNN-LSTM model predicted LVH by 12-lead ECG with an AUC of 0.66 (sensitivity 72%, specificity 60%) for male patients, which performed better than that for female patients (AUC: 0.59, sensitivity 50%, specificity 71%).

**Conclusion:**

Our study established a CNN-LSTM model to diagnose LVH by 12-lead ECG with higher sensitivity than current ECG diagnostic criteria. This CNN-LSTM model may be a simple and effective screening tool of LVH.

## Introduction

Left ventricular hypertrophy (LVH) is an early structural and functional cardiac change of hypertension, with an estimated echocardiographic prevalence of 36–41% (1). Other causes of LVH include aortic stenosis, hypertrophic cardiomyopathy, valvular heart disease, infiltrative heart muscle disease, storage and metabolic disorders (2, 3). The incidence of LVH is further affected by age and obesity (4, 5). Previous studies showed that LVH is an independent risk of arrhythmias (3), heart failure (6) and mortality (7).

Echocardiography is the current standard diagnostic method (8), whereas 12-lead ECG is the most commonly used diagnostic tool in clinical cardiology as it allows a rapid screening of LVH. However, current ECG criteria of LVH including the Cornell voltage and the Sokolow-Lyon voltage criteria have low sensitivity (7, 9). These criteria mainly focus on increased QRS complex amplitude, but overlook a leftward shift of electrical axis in the frontal plane, ST segment deviation and T wave changes, which are also principal ECG diagnostic characteristics for LVH (10). Besides, the interpretation of these ECG criteria are tedious for doctors, affecting the efficiency and accuracy of diagnosis. To improve these limitations of the current ECG criteria for LVH, new methods for analysis of ECG are urgently needed.

Since the digitalization of ECG, artificial intelligence methods have been employed in computerized interpretation of ECGs (11). Recently, few studies were presented by machine learning for the ECG characteristics to detect presence of LVH (12, 13). Among these methods, deep learning (DL) techniques are superior to conventional machine learning techniques due to its ability of automatic feature extraction. The Convolutional Neural Network (CNN), combined with the Long Short-Term Memory (LSTM) model, appear to be the most useful architectures for classification (14). A 16-layer CNN-LSTM model was efficaciously used to classify coronary atherosclerotic disease (CAD), myocardial infarction, and chronic heart failure signals, with a precision rate of 98.5% (15). Our previous study also showed that the CNN-LSTM performed better than the CNN, LSTM, and doctors in detecting acute ST-segment elevation myocardial infarction (STEMI) based on 12-lead ECG, with an area under the curve (AUC) of 0.99 (16). Accordingly, our study aimed to establish a DL model based on the CNN-LSTM for reliable and rapid detection of LVH using 12-lead ECG.

## Methods

### Study population

A total of 3,120 patients hospitalized at the Third Affiliated Hospital of Sun Yat-sen University in China from January 2017 to December 2019 were recorded. Only the first admission for each patient was included; repeated hospitalizations were not evaluated in this study. Finally, 1,863 patients with ECG obtained within 1 week after hospitalization were included for analysis. Exclusion criteria were as follows: complete left or right bundle branch block, ventricular paced rhythm, ventricular arrhythmia at the time of ECG acquisition. Another independent cohort consisted of 453 patients was used as the internal test set 2 using the same inclusion and exclusion criteria. All personal details were erased to protect the confidentiality of patients' data. Data collection was approved by the ethics committee at the Third Affiliated Hospital of Sun Yat-sen University.

### Baseline data collection

Data was extracted from the standard clinical electronic medical record (EMR) database of the Third Affiliated Hospital of Sun Yat-sen University, including demographic characteristics, comorbidities, laboratory tests, and medicines. The comorbidities were retrieved according to ICD-10 diagnostic codes.

### Acquisition and procession of echocardiography data

Comprehensive 2-dimensional Doppler echocardiography, the gold standard to assess LVH, was routinely performed using commercially available ultrasound equipment. Acquisitions and measurements were performed by two experienced cardiac ultrasound doctors. LVH is defined as a left ventricular mass index (LVMI) >115 g/m^2^ in male subjects and >95 g/m^2^ in female subjects (17). Calculation of relative wall thickness (RWT) with the formula (2× posterior wall thickness)/(LV internal diameter at end-diastole), permits categorization of an increase in LV mass as either concentric (RWT > 0.42) or eccentric (RWT ≤ 0.42) hypertrophy (17).

### Acquisition and procession of ECG data

ECG was performed at a sampling rate of 1,000 Hz, and acquired in the supine position using the ECGNET Vision 3.0 (SanRui Electronic Technology, Guangdong, China). The ECG signal had to be clear, stable baseline with no interference. All ECG data were labeled with the study ID, and stored as XML file format following the H7L standard on a secure server. The quality of ECG data and ECG interpretations were independently reviewed by 2 cardiologists. The comparison of our model was referred to the Cornell voltage criteria and the Sokolow-Lyon voltage, given their relative higher sensitivity and specificity (9, 18). The sex-specific Cornell voltage criteria was computed as the amplitude of R in aVL plus the amplitude of S or QS complex in V3 (RaVL + SV3) with a cutoff of >2.8 mV in men and >2.2 mV in women. The Sokolow-Lyon voltage was obtained by adding the amplitude of S in V1 and the amplitude of R in V5 or V6 ≥3.5 mV (SV1 + RV5 or RV6) (19).

## Deep-learning modeling

### ECG data extraction

ECG data was extracted from XML files, consisted of 12 channels. The duration of ECG generally lasted from 10 to 90 s, and were cut into 5-s segment. The specification of each ECG segment was finally intercepted (5,000, 12), which was then utilized in the input model.

### Data balance

There was imbalance in the quantity of cases and ECG segments between the control and LVH groups, as the latter group had less cases and ECG segments. To solve this problem, we drew sample cases and ECG segments of the control group referring to these of the LVH group, at last the cases and ECG segments were balanced in two groups.

The model was evaluated through 5-fold cross-validation technique. In each repetition of the cross-validation process, one part was selected as the validation set, another part was selected as the test set, while the remaining parts were served as the training set. Thus, the datasets of cases and ECG segments were needed to be equally split into 5 parts following below steps: (1) ECG segments of each case were ranked by number; (2) counted the frequency of the number of ECG segments; (3) if the ECG data of cases had the same quantity of segments and the number of those cases was more than 5, the ECG data of those five cases were selected and evenly divided into five parts, and then the remaining ECG data of cases were partitioned into five proximately equal parts, making the total number of cases and ECG segments among 5-fold subsets approximate. In order to split five equal parts rapidly, we developed an algorithm replacing manual processing with automation. The final dataset included 36,350 ECG segments (*n* = 931) and 36,348 ECG segments (*n* = 932) in the control and LVH groups, respectively. Previous studies have showed that the sensitivity and specificity of ECG criteria could be influenced by gender and left ventricular geometry, therefore we performed subgroup analyses. And we also balanced data for all subgroup analyses using the same method.

### Model architecture and training

The architecture of CNN-LSTM model has been described in our previous study (16). In the training process, the model input was 12-lead ECG segment which had the specification of (5,000, 12). The first part of the CNN-LSTM model was CNN layers. The (5,000, 12) ECG segments were split into m smaller segments (length of smaller segments = 5,000/m) to train m CNN time Distributed layers simultaneously. The time Distributed layer is fully connected in the time dimension. In CNN time Distributed layer, weight parameters or convolution kernels were shared, instead of each have its own weight. We made the number of smaller segments (m) as a parameter with a value scope in (1, 2, 5, 10, 20, 25, 50, 100, 200, 250, and 500). The number of smaller segment (m) was settled according to the best validation output during training process. The number of CNN layers ranged from 1 to 5, and that of LSTM layers was 2. The CNN layer kernels would be selected from the scope of (16, 24, 32, 48, 56, and 64). All hyper-parameters would be Grid Search by keras tuner tool, which could automatically record and compare the accuracy of different models. Finally, the model with the best performance and corresponding hyper-parameters were selected, and then the parameters of the best model were utilized for LVH prediction based on ECG. Among all of the models explored, the CNN-LSTM model which had 200 smaller segments to input data, and contained 3 CNN layers (16 kernels in each layer), followed by 2 LSTM layers (200 LSTM units and 2 LSTM units in each LSTM layer, respectively) performed the best. The last 2 LSTM units output was predictive probability of the control and LVH groups. The DL models of each subgroup, including gender and RWT, were developed in the same process. More details of all models were showed in Supplementary Table 1.

### Statistical analysis

The baseline characteristics were described as mean (standard deviation) (SD) or median with interquartile range (IQR) for continuous variables, and categorical variables were described as proportions. Kolmogorov-Smirnov test was used for continuous variables whether conforming to normal distribution. Differences in baseline characteristics were compared using *t*-test between two groups or analysis of variance (ANOVA) for continuous variables, while Mann-Whitney *U*-test between two groups and Kruskal-Wallis test among three groups was for abnormal distribution, and Chi-square test was applied for categorical variables. The statistical analyses were performed using the SPSS 22.0. A 2-tailed *P*-value < 0.05 was considered statistically significant.

Receiver operating characteristic (ROC) curve analysis and area under the curve (AUC) were used to evaluate the diagnostic efficacy of CNN-LSTM models and conventional ECG indexes of LVH. Delong's test was used to compare the performance of two ROC curves. Sensitivity, specificity, positive predictive value (PPV), negative predictive value (NPV) and F1-score were calculated with python (version 3.6.9).

## Results

### Study population and baseline characteristics

**Figure 1:**
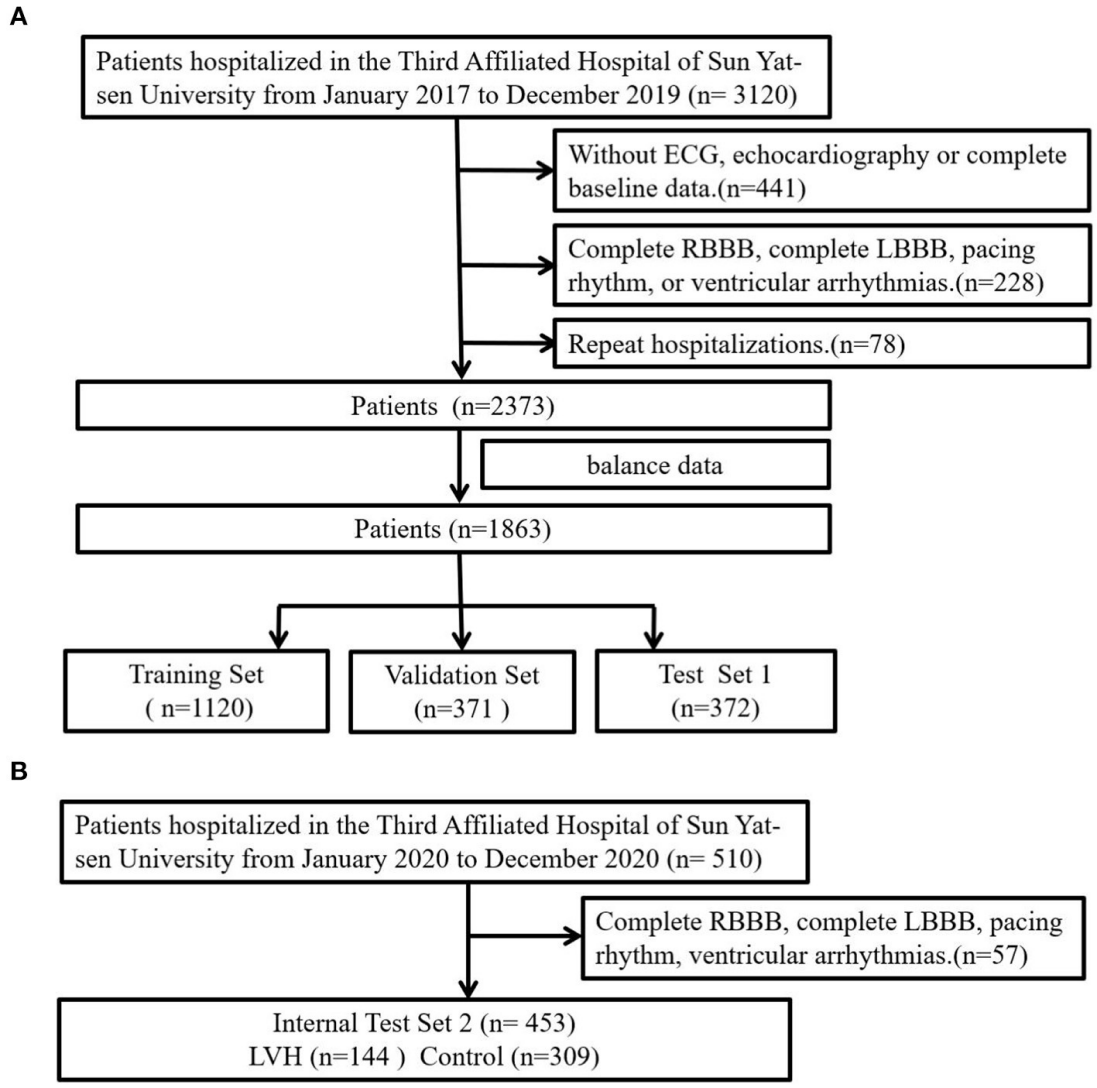
Study flow diagram. **(A)** The first cohort was further divided into training, validation and test sets. **(B)** The second cohort was used as internal test 2 to evaluate developed DL model.

The study flow chart was shown in Figure 1. In the first cohort, 1,863 patients were divided into 2 groups according to the LVMI criteria: LVH group (*n* = 932) and control group (*n* = 931). Compared with the patients of control, patients in the LVH group were older, composed of higher proportion of female, more combined with hypertension, chronic heart failure (CHF), chronic kidney disease (CKD), as well as more likely to receive angiotensin-converting enzyme inhibitor (ACEI) and diuretics, but had lower level of hemoglobin (HGB). More details were shown in Table 1.

**Table 1:** Patient characteristics between LVH and control groups.

**Characteristics**	**LVH**	**Control**	***P*** **-value**
	**(*n* = 932)**	**(*n* = 931)**	
**Demographic**			
Female, *n* (%)	511 (54.8)	273 (29.3)	<0.001
Age, years	67.3 (10.5)	63.9 (11.3)	<0.001
**Medical history**			
CAD, *n* (%)	601 (64.5)	564 (60.6)	0.082
HT, *n* (%)	586 (62.9)	486 (52.2)	<0.001
CHF, *n* (%)	330 (35.4)	223 (24.0)	<0.001
DM, *n* (%)	306 (32.8)	333 (35.8)	0.182
Stroke, *n* (%)	129 (13.8)	122 (13.1)	0.641
CKD, *n* (%)	85 (9.1)	47 (5.0)	0.001
STEMI, *n* (%)	23 (2.5)	16 (1.7)	0.259
**Laboratory examination**			
HDL-C (mmol/L)	1.07 (0.29)	1.04 (0.27)	0.270
LDL-C (mmol/L)	2.77 (1.06)	2.82 (1.03)	1.030
HGB (g/L)	125.71 (19.06)	133.02 (18.74)	<0.001
PLT (10∧9/L)	230.52 (85.34)	228.64 (68.03)	0.607
BUN (mmol/L)	6.82 (4.58)	6.16 (2.70)	<0.001
Cr (umol/L)	103.92 (122.34)	87.81 (58.00)	<0.001
UA (umol/L)	394.94 (124.77)	394.92 (112.51)	0.997
potassium (mmol/L)	3.99 (0.45)	4.00 (0.40)	0.620
sodium (mmol/L)	141.58 (3.24)	141.39 (5.17)	0.767
**ECG**			
RV5 (mV)	1.49 (1.12, 1.99)	1.40 (1.09, 1.75)	<0.001
RV6 (mV)	1.20 (0.87, 1.58)	1.10 (0.86, 1.42)	<0.001
RaVL (mV)	0.42 (0.24, 0.63)	0.33 (0.17, 0.54)	<0.001
SV1 (mV)	−0.81 (−1.12 to −0.53)	−0.72 (−0.97 to −0.50)	<0.001
SV3 (mV)	−0.93 (−1.32 to −0.58)	−0.86 (−1.17 to −0.55)	0.002
Cornell voltage LVH, *n* (%)	414 (45.2)	240 (26.3)	<0.001
Sokolow-Lyon LVH, *n* (%)	109 (11.9)	18 (2.0)	<0.001
**Echocardiography**			
LVEF (%)	64.08 (9.35)	67.43 (5.17)	<0.001
LVEDD (mm)	49.23 (5.34)	44.6 (3.97)	<0.001
LVPW (mm)	10.44 (1.10)	9.58 (1.01)	<0.001
IVS (mm)	11.77 (1.68)	10.50 (1.34)	<0.001
LVMI (g/m^2^)	129.28 (28.93)	89.97 (14.47)	<0.001
Concentric LVH, *n* (%)	515 (55.3)	532 (57.1)	0.412
**Treatment**			
ACEI, *n* (%)	192 (20.6)	113 (12.1)	<0.001
ARB, *n* (%)	264 (28.3)	234 (25.1)	0.120
Spirolactone, *n* (%)	134 (14.4)	107 (11.5)	0.064
CCB, *n* (%)	366 (39.3)	326 (35.0)	0.057
BB, *n* (%)	586 (62.9)	563 (60.5)	0.286
Diuretics, *n* (%)	240 (25.8)	186 (20.0)	0.003

CAD, coronary artery disease; HT, hypertension; DM, diabetes mellitus; CHF, chronic heart failure; CKD, chronic kidney disease; STEMI, ST-segment elevation myocardial infarction; HDL-C, high density lipoprotein cholesterol; LDL-C, low density lipoprotein cholesterol; HGB, hemoglobin; PLT, platelet; BUN, blood urea nitrogen; Cr, creatinine; UA, uric acid; LVEF, left ventricular ejection fraction; LVEDD, left ventricular end-diastolic dimension; LVPW, left ventricle posterior wall; IVS, ventricular septum; LVMI, left ventricular mass index; ACEI, angiotensin-converting enzyme inhibitor; ARB, angiotensin receptor blocker; CCB, calcium channel blocker; BB, beta-block.

Further, patients in the first cohort were evenly split into 3 sets: the training set (*n* = 1,120), the validation set (*n* = 371) and the test set 1 (*n* = 372). Patients in the training set had higher prevalence of CAD. There were no significant difference in other clinical characteristics among the three sets. More baseline characteristics were summarized in Supplementary Table 2. In the internal test set 2, patients in the LVH group were older, composed of higher proportion of female and had higher prevalence of CHF, but had lower level of HGB (Supplementary Table 3).

### The predictive value of DL models in LVH diagnosis

**Figure 2:**
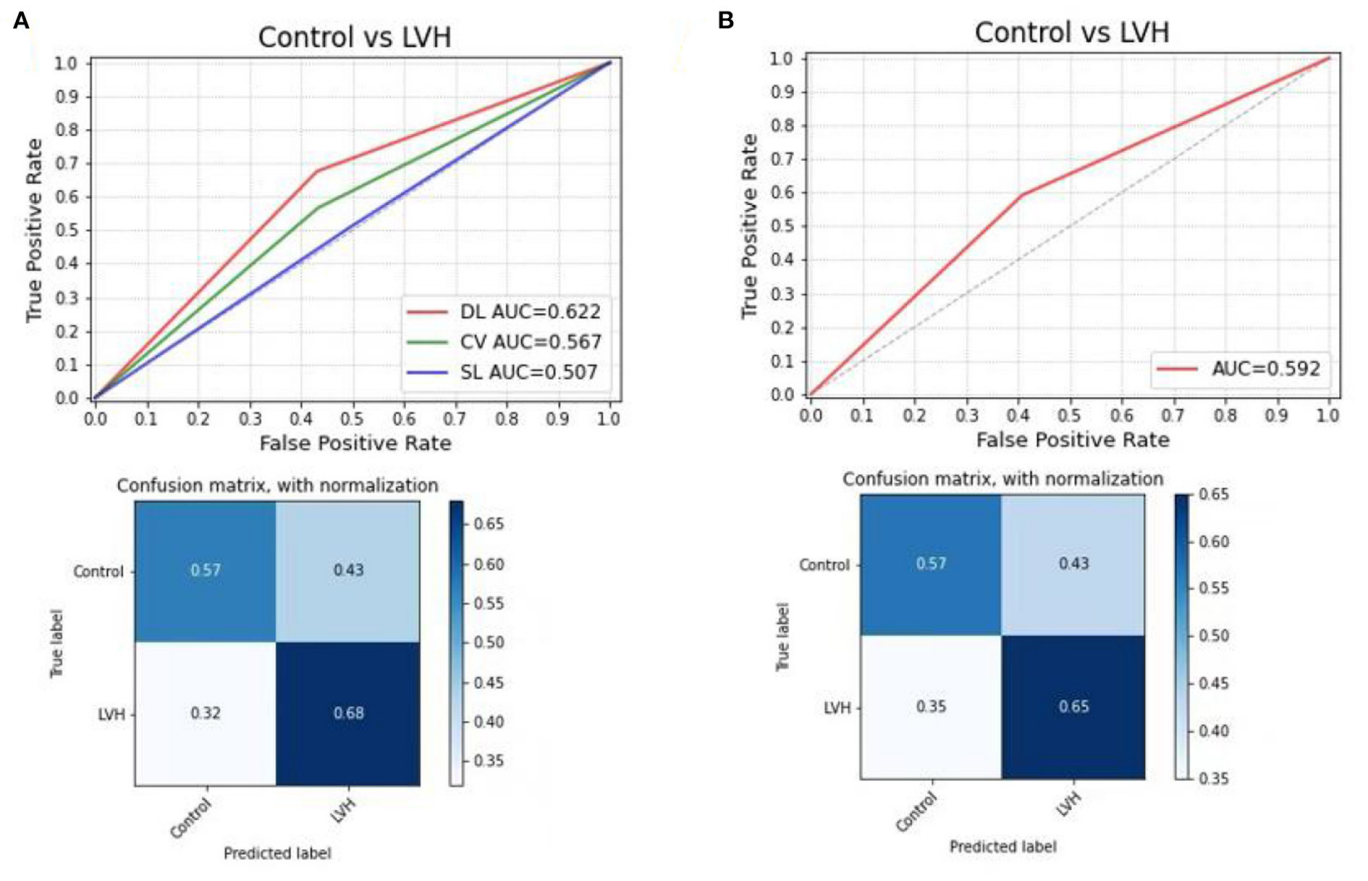
Receiver operating characteristic curve analysis, **(A)** compared the DL model with Cornell voltage and Sokolow-Lyon voltage in test set 1, the confusion matrix for predicting control and LVH using the DL model in the test set 1; **(B)** to test the DL model in internal test set 2. DL, deep learning model; CV, Cornell voltage, SL, Sokolow-Lyon voltage.

LVH was predicted by the CNN-LSTM model with an AUC of 0.62 (sensitivity 68%, specificity 57%) in the test set 1, which had a better performance than the Cornell voltage criteria (AUC: 0.57, sensitivity 48%, specificity 72%) and the Sokolow-Lyon voltage (AUC: 0.51, sensitivity 14%, specificity 96%). Differences in ROC curves were statistically compared *via* Delong's test (CNN-LSTM model vs. Cornell voltage criteria, *p*-value = 0.075; CNN-LSTM model vs. Sokolow-Lyon, *p*-value = 0.037). Although no significant difference was found between CNN-LSTM model and Cornell voltage criteria, the sensitivity of CNN-LSTM model was higher than that of Cornell voltage criteria. In the internal test set 2, the CNN-LSTM model had a stable performance in predicting LVH with an AUC of 0.59 (sensitivity 65%, specificity 57%) (Figure 2), which was comparable to that of the internal test set 1.

**Figure 3:**
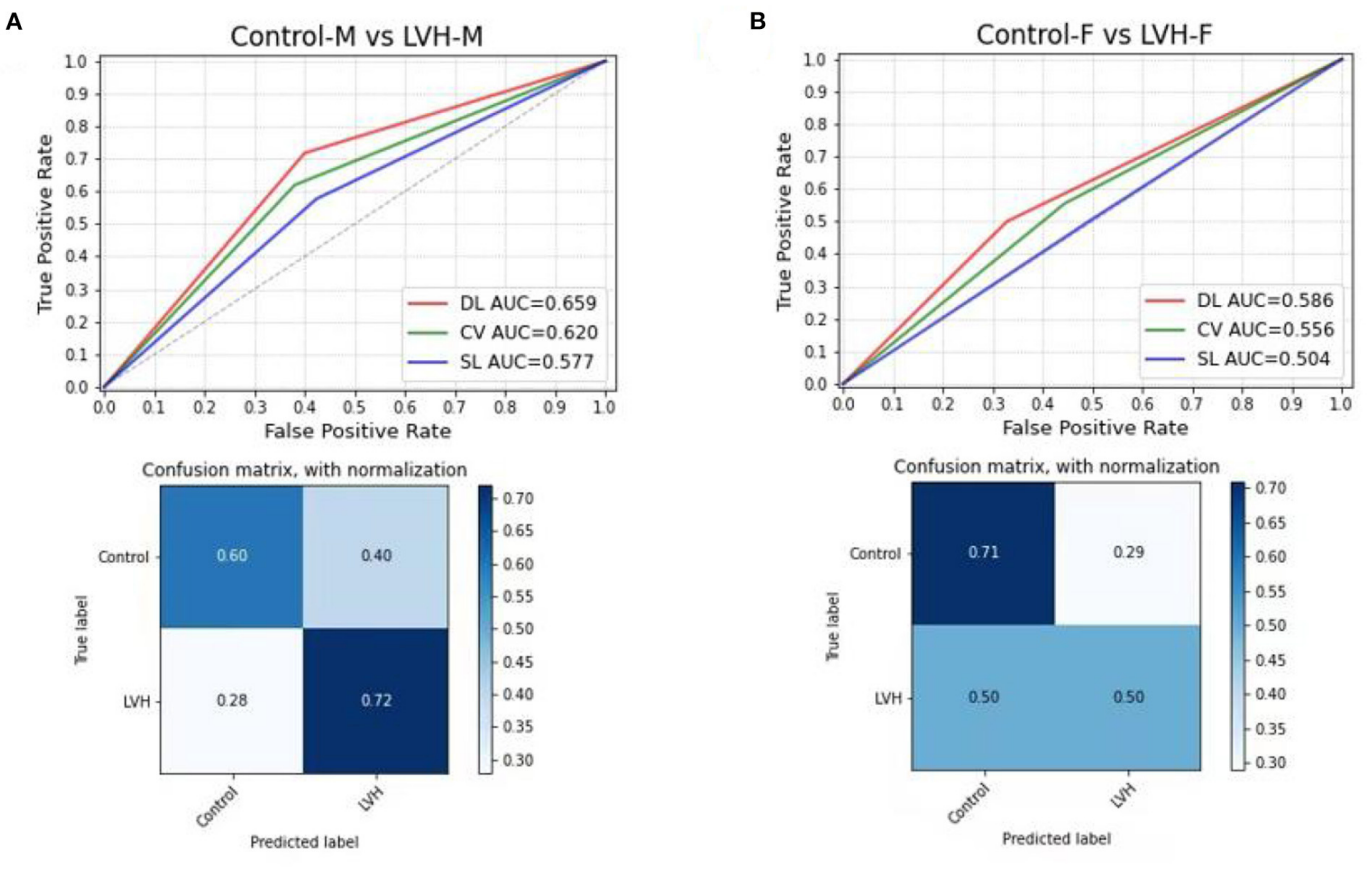
Comparing the DL model with Cornell voltage and Sokolow-Lyon voltage to predict LVH, the confusion matrix for predicting control and LVH using the DL model in the test set; **(A)** for male patients; **(B)** for female patients. DL, deep learning model; CV, Cornell voltage; SL, Sokolow-Lyon voltage.

**Figure 4:**
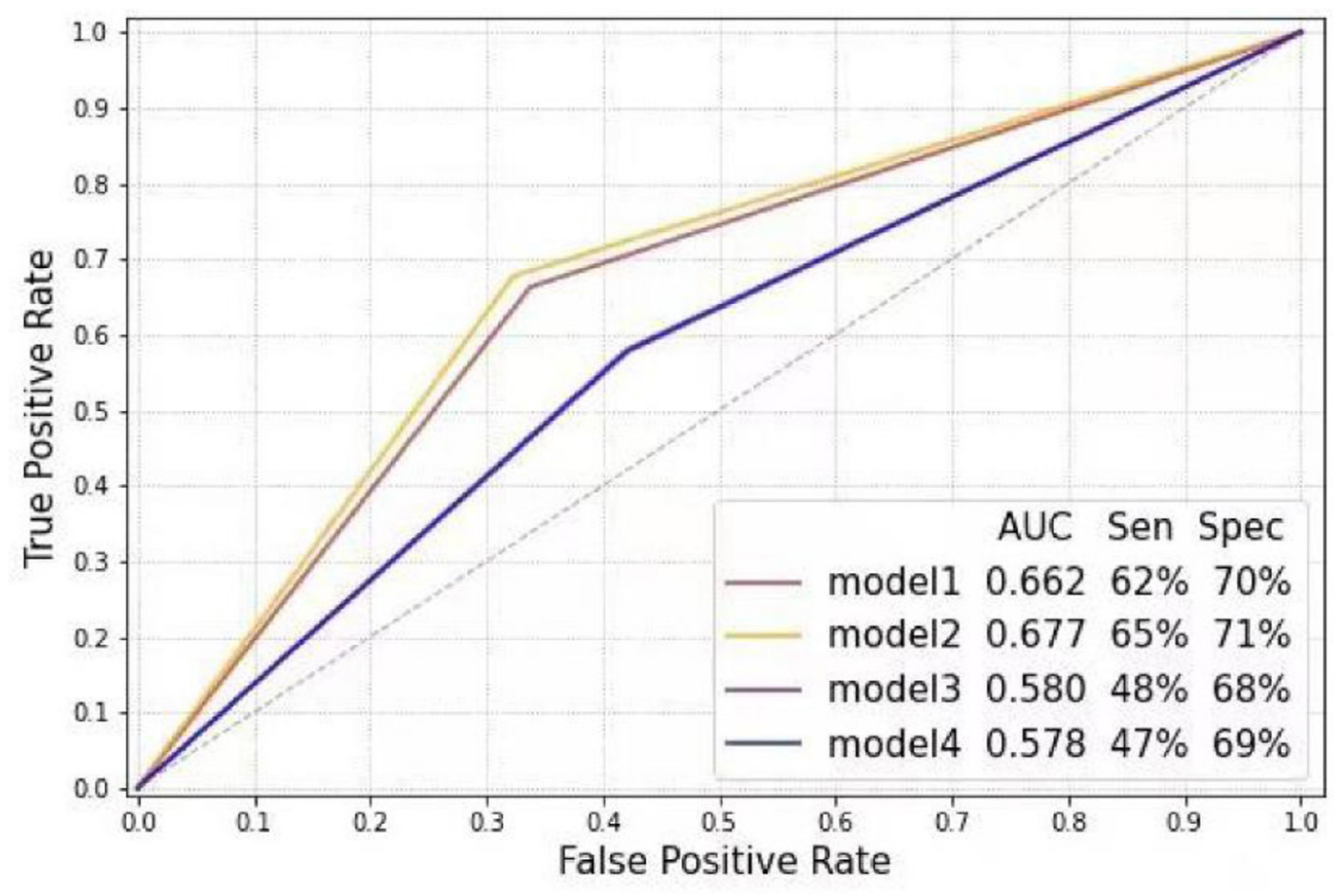
Receiver operating characteristic curve analysis of different models according to gender and relative wall thickness (Model 1: Control-M vs. concentric LVH-M; Model 2: Control-M vs. eccentric LVH-M; Model 3: Control-F vs. concentric LVH-F; Model 4: Control-F vs. eccentric LVH-F). LVH-F, female patients with left ventricular hypertrophy; LVH-M, male patients with left ventricular hypertrophy; Control-F, female patients in control group; Control-M, male patients in control group.

In the subgroup analysis, the first step was to train different DL models according to gender. In the test sets, the CNN-LSTM model predicted LVH with an AUC of 0.66 (sensitivity 72%, specificity 60%) for male patients, which was better than that for female patients (AUC: 0.59, sensitivity 50%, specificity 71%) (Figure 3). The second step was to evaluate the effect of left ventricular geometry on the diagnosis of ventricular hypertrophy based on ECG. The DL models were trained for concentric and eccentric hypertrophy according to RWT. In the test sets, the CNN-LSTM model predicted concentric hypertrophy with an AUC of 0.66 (sensitivity 62%, specificity 70%) and eccentric hypertrophy with an AUC of 0.68 (sensitivity 65%, specificity 71%) in male patients, and an AUC of 0.58 (sensitivity 48%, specificity 68%) for concentric hypertrophy and an AUC of 0.58 (sensitivity 47%, specificity 69%) for eccentric hypertrophy in female patients (Figure 4) (Supplementary Table 4).

## Discussion

This is a study to develop DL models of LVH diagnosis based on a large real-world ECG database. Our main achievement was that we built a DL model based on CNN-LSTM with higher sensitivity than current ECG diagnostic criteria. Moreover, we constructed different CNN-LSTM models to predict LVH for male and female patients separately, and the predictive value was better in male patients.

Our DL model predicted LVH with higher sensitivity than the Cornell voltage criteria and Sokolow-Lyon voltage (68, 48, and 14%, respectively), whereas its specificity was inferior to these two criteria (57, 72, and 96%, respectively). The accuracy of our model still needed to be improved. In the study of Bressman et al., found that the sensitivity and specificity of ECG for left ventricular hypertrophy were 30.7 and 84.4% in a cohort of 13,960 subjects using a computer-generated algorithm, which is similar to the combination of the Sokolow-Lyon and Framingham criteria (20). Peguero et al. proposed a new ECG criteria involved measuring the amplitude of the deepest S wave (SD) in any single lead and adding it to the S wave amplitude of lead V4 (SV4), which outperformed Cornell voltage with a significantly higher sensitivity (62 vs. 35%) in a relatively small sample size (21). However, another study found that the Cornell voltage carried the best AUC of 0.678 (sensitivity 33.1%, specificity 88.8%), while Peguero Lo Presti criterion had an AUC of 0.64 (sensitivity 42.3%, specificity 75.8%) in a cohort of 2,134 patients (19). Current ECG criteria of LVH have low sensitivity, limit the application of ECG in screening for LVH. Recently, a few studies utilized machine learning techniques for ECG and clinical characteristics to diagnose LVH. Lin et al. used a support vector machine classifier as the machine learning method for 31 clinical characteristics and 28 ECG parameters to detect LVH, successfully achieving a specificity of 73.3%, and a much better sensitivity of 86.7%, compared to 3.3 and 52.7% of the Cornell and Sokolow-Lyon voltage criteria in a large sample of 2,196 males (12). Although this research developed a method with high accuracy in a large sample size, the patients included were only of younger males, and this model needed lots of clinical characteristics. Additionally, a machine-learning technique called Bayesian Additive Regression Trees was developed to predict LVH based on ECG and participant characteristics, and the result showed a specificity more than 93% but a poor sensitivity of only 29.0% in a cohort of 4,714 participants from the Multi-Ethnic Study of Atherosclerosis study (13). Khurshid et al. trained a CNN to predict cardiac magnetic resonance (CMR)-derived LV mass using 12-lead ECGs (LVM-AI) in the UK Biobank prospective cohort of 32,239 individuals. The results showed that the LVH discrimination of LVM-AI was 0.653 (sensitivity 34%, specificity 96%) and 0.621 (sensitivity 41%, specificity 83%) in the independent UK Biobank test set and Mass General Brigham, respectively. However, low sensitivity was still limiting the application of these models. On the other hand, the CNN-LSTM was able to detect CAD ECG signals with a diagnostic accuracy of 99.85% with blind-fold strategy (22). Our previous study showed the ECG DL diagnosis systems based on the CNN-LSTM have a good performance to detect STEMI and predict culprit vessel occlusion (16). On this basis, we developed a DL model of LVH diagnosis that showed higher sensitivity than current ECG criteria.

Moreover, previous work showed that female gender was associated with lower sensitivity but higher specificity (20, 23). Consistently, in our study, the LVH diagnosed by the DL model was lower in female patients (50% sensitivity, 71% specificity), compared to 72% of sensitivity and 60% of specificity for male patients. Additionally, left ventricular geometry is associated with ECG-defined left ventricular hypertrophy (24). An RWT > 0.42 demonstrated an increased sensitivity and decreased specificity for LVH (20). However, our models showed similar sensitivity to predict eccentric and concentric hypertrophy in female patients, and even higher sensitivity for eccentric hypertrophy in male patients.

There are some advantages in our DL models based on CNN-LSTM. First, the most common method of model training is to manually set a parameter, and the optimal value is selected after repeated experiments, which is inconvenient for clinical application. In our model training stage, the grid search method was used to search all possible parameter combinations of each model. For each parameter, grid search algorithm can extensively search the whole possible parameters space, and these parameters searching can be done in parallel, regardless of computing resource constraints, to reduce the training time. Moreover, our DL model did not need additional preprocessing for ECG data like removing noise, which may also perform well with different sources of ECG. However, the accuracy of our model still needed to be improved. Previous study showed multiple patient characteristics were associated with differences in sensitivity and specificity of LVH prediction by ECG. Therefore, adding the baseline characteristics like age, gender, body mass index, comorbidities to our model training may improve its performance. On the other hand, the attention module integrates channel information, obtains the importance of features and allocates attention weight to make the network pay attention to important features, so channel-wise attention could be added to different convolution layers in order to optimize the CNN-LSTM model. In terms of clinical application, our DL model was established in a real-world ECG database, in which all patients were included regardless of the admitting diagnosis. In addition, LVH is a modifiable risk factor related to systolic BP and regression of LVH may reduce subsequent CV events (25). Therefore, it might be helpful in the better management of hypertension. Besides, our CNN-LSTM model is an end-to-end approach, it only utilized raw ECG data input and built binary classification and multiclassification without experts or experienced cardiologists. It could be able to give primary diagnosis timely and reduce the workload of doctors.

### Limitations

Some limitations of our study should be considered. Our study was a single-center study, the models may have the risk of generalizing poorly to other hospital systems and other datasets. Besides, our ECG diagnostic models based on CNN-LSTM have higher sensitivity at the expenses of relatively lower specificity compared to currently commonly used ECG diagnostic criteria. But ECG used as a screening tool, the interpretation method with higher sensitivity is more likely to identify more individuals with LVH who need confirmation of the diagnosis with echocardiography or MRI. Moreover, this study population mainly included south China population. Therefore, more researches from different regions and ethnic groups are necessary to confirm these findings.

## Conclusion

Our ECG diagnostic model based on the CNN-LSTM has higher sensitivity than currently used ECG diagnostic criteria. The performance of the model trained for male patients was better than that for female patients. Therefore, this CNN-LSTM model may be a simple and effective screening tool of LVH in hypertensive patients and general population.

## Data availability statement

The original contributions presented in the study are included in the article/Supplementary material, further inquiries can be directed to the corresponding author/s.

## Ethics statement

All traceable personal identifiers were removed from the analytic dataset to protect patients' privacy. The study protocol was approved by the Third Affiliated Hospital, Sun Yat-sen University ethics committee and the study was performed according to the declaration of Helsinki.

## Author contributions

XZ, GH, and LW: research idea and study design. MW, J-RW, BZ, YeL, XY, SL, BT, and LL: data acquisition. DL and YoL: data analysis/interpretation. XZ and GH: statistical analysis. XQ, LH, and XX: supervision and mentorship. XQ, LH, XX, SQ, YC, and XH: writing guidance. Each author contributed important intellectual content during manuscript drafting or revision and accepts accountability for the overall work by ensuring that questions on the accuracy or integrity of any portion of the work are appropriately investigated and resolved. All authors read and approved the final version.

## Funding

This study received funding from Guangdong Medical Research Foundation (A2019079), the National Natural Science Foundation of China (81770826 and 81370447), Science and Technology Planning Project of Guangdong Province (2016A050502014), National Key R&D Program (2018yfc1705105 and 2017YFA0105803), the 5010 Clinical Research Projects of Sun Yat-sen University (2015015), the Key Area R&D Program of Guangdong Province (2019B020227003), and the Science and Technology Plan Project of Guangzhou City (202007040003). The funders were not involved in the study design, collection, analysis, interpretation of data, the writing of this article or the decision to submit it for publication.

## Conflict of interest

Authors XZ, LW, XX, and XQ work in the Third Affiliated Hospital of Sun Yat-sen University, GH was employed by China Unicom (Guangdong) Industrial Internet Ltd., SQ works for China Unicom (Guangdong) Industrial Internet Ltd., LH works in Research Institute of Tsinghua, Pearl River Delta. Author J-RW was employed by LCFC (Hefei) Electronics Technology Co., Ltd., Hefei LCFC Information Technology Co., Ltd. The remaining authors declare that the research was conducted in the absence of any commercial or financial relationships that could be construed as a potential conflict of interest.

## Publisher's note

All claims expressed in this article are solely those of the authors and do not necessarily represent those of their affiliated organizations, or those of the publisher, the editors and the reviewers. Any product that may be evaluated in this article, or claim that may be made by its manufacturer, is not guaranteed or endorsed by the publisher.
