# Supplementary material for: Deep learning assessment of left ventricular hypertrophy based on electrocardiogram
Source: Front Cardiovasc Med. 2022 Aug 11;9:952089. doi: 10.3389/fcvm.2022.952089 (PMC9406285; doi:10.3389/fcvm.2022.952089)
Supplement: Supplementary file 1 [file Data_Sheet_1.docx]

**Supplementary Table 1 Network structure parameters of CNN-LSTM models**

|  | CNN timeDistributed layers (m) | number of CNN layers | kernels in CNN layer | units in first LSTM  layer | units in second LSTM layer |
| --- | --- | --- | --- | --- | --- |
| Control vs LVH | 200 | 3 | 16,16,16 | 200 | 2 |
| Control-F vs LVH-F | 50 | 3 | 16,16,16 | 50 | 2 |
| Control-M vs LVH-M | 50 | 3 | 16,16,16 | 50 | 2 |
| Control-F vs concentric LVH-F | 100 | 2 | 16,16 | 100 | 2 |
| Control-F vs eccentric LVH-F | 25 | 3 | 16,16,16 | 250 | 2 |
| Control-M vs concentric LVH-M | 10 | 3 | 16,16,16 | 10 | 2 |
| Control-M vs eccentric LVH-M | 250 | 3 | 16,16,16 | 250 | 2 |

**Abbreviation:** CNN= convolutional neural network; LSTM= long short-term memory; LVH= left ventricular hypertrophy; LVH-F= female patients with left ventricular hypertrophy; LVH-M= male patients with left ventricular hypertrophy; Control-F= female patients in control group; Control-M= male patients in control group;

**Supplementary Table 2 Patient characteristics among Training, Validation and Test sets**

| **Characteristics** | Training set  （n=1120） | Validation set  (n=371) | Test set 1  (n=372) | P value |
| --- | --- | --- | --- | --- |
| **Demographic** | | | | |
| female, n (%) | 458 (40.9) | 157 (42.3) | 169 (45.4) | 0.306 |
| Age，years | 65.5 (10.9) | 65.5 (11.2) | 66.1 (11.2) | 0.595 |
| **Medical history** | | | | |
| CAD, n (%) | 733 (65.4) | 210 (56.6) | 222 (59.7) | 0.004 |
| HT, n (%) | 636 (56.8) | 221 (59.6) | 215 (57.8) | 0.639 |
| DM, n (%) | 378 (33.8) | 117 (31.5) | 144 (38.7) | 0.099 |
| CHF, n (%) | 339 (30.3) | 99 (26.7) | 115 (30.9) | 0.359 |
| Stroke, n (%) | 155 (13.8) | 46 (12.4) | 50 (13.4) | 0.780 |
| CKD, n (%) | 82 (7.3) | 31 (8.4) | 19 (5.1) | 0.200 |
| STEMI, n (%) | 25 (2.2) | 7 (1.9) | 7 (1.9) | 0.876 |
| **Laboratory examination** | | | | |
| HDL-C (mmol/L) | 1.06 (0.28) | 1.05 (0.28) | 1.07 (0.29) | 0.590 |
| LDL-C (mmol/L) | 2.81 (1.07) | 2.81 (0.97) | 2.74 (1.05) | 0.480 |
| HGB (g/L) | 129.33 (18.99) | 130.72 (19.46) | 128.149 (19.7) | 0.199 |
| PLT (10^9/L) | 228.60 (77.82) | 229.43 (79.85) | 232.64 (72.59) | 0.689 |
| BUN (mmol/L) | 6.52 (3.52) | 6.30 (2.81) | 6.86 (5.13) | 0.123 |
| Cr (umol/L) | 96.23 (93.22) | 88.75 (59.23) | 102.01 (128.76） | 0.175 |
| UA (umol/L) | 395.82 (118.40) | 385.60 (106.2) | 401.29 (130.53） | 0.202 |
| potassium (mmol/L) | 3.99 (0.42) | 4.00 (0.42) | 3.98（0.44） | 0.914 |
| sodium (mmol/L) | 141.58 (4.79) | 141.39 (3.55) | 141.41（3.38） | 0.698 |
| ECG | | | | |
| RV5 (mV) | 1.44 [1.08,1.83] | 1.47 [1.10, 1.95] | 1.46 [1.12, 1.85] | 0.317 |
| RV6 (mV) | 1.14 [0.85, 1.49] | 1.17 [0.85, 1.50] | 1.14 [0.90, 1.47] | 0.780 |
| RaVL (mV) | 0.39 [0.20, 0.58] | 0.38 [0.20, 0.60] | 0.37 [0.18, 0.55] | 0.291 |
| SV1 (mV) | -0.75 [-1.03, -0.51] | -0.80 [-1.08, -0.55] | -0.75 [-1.04, -0.50] | 0.138 |
| SV3 (mV) | -0.89 [-1.24, -0.55] | -0.89 [-1.24, -0.55] | -0.86 [-1.28, -.059] | 0.844 |
| Cornell voltage LVH, n (%) | 364 (33.2) | 153 (42.1) | 138 (37.6) | 0.004 |
| Sokolow-Lyon LVH, n (%) | 67 (6.1) | 29 (8.0) | 33 (9.0) | 0.180 |
| **Echocardiography** | | | | |
| LVEF (%) | 65.58 (7.98) | 66.08 (7.85) | 65.95 (7.48) | 0.492 |
| LVEDD (mm) | 46.94 (5.38) | 47.10 (5.25) | 46.53 (4.79) | 0.449 |
| LVPW (mm) | 10.00 (1.34) | 9.97 (1.21) | 10.07 (1.08) | 0.521 |
| IVS (mm) | 11.14 (1.65) | 11.16 (1.78) | 11.11 (1.51) | 0.934 |
| LVMI (g/m^2^) | 109.93 (30.61) | 109.29 (29.36) | 109.07 (30.16) | 0.876 |
| Concentric LVH, n (%) | 630 (56.3) | 197 (53.1) | 220(59.1) | 0.252 |
| **Treatment** | | | | |
| ACEI, n (%) | 189 (16.9) | 60 (16.2) | 56 (15.1) | 0.708 |
| ARB, n (%) | 301 (26.9) | 95 (25.6) | 102 (27.4) | 0.843 |
| [spirolactone](javascript:;), n (%) | 150 (13.4) | 47 (12.7) | 44 (11.8) | 0.727 |
| CCB, n (%) | 416 (37.1) | 137 (36.9) | 139 (37.1) | 0.992 |
| BB, n (%) | 676 (60.4) | 236 (63.6) | 237 (63.7) | 0.356 |
| [diuretic](javascript:;), n (%) | 260 (23.2) | 86 (23.2) | 80 (21.5) | 0.783 |

Abbreviations are shown as Table 1

**Supplementary Table 3 Patient characteristics of the internal test 2 cohort**

| **Characteristics** | LVH group  (n=144) | Control group  (n=309) | P value |
| --- | --- | --- | --- |
| **Demographic** | | | |
| female, n (%) | 81 (56.3) | 93 (28.2) | <0.001 |
| Age，years | 67.2 (10.5) | 64.4 (10.5) | 0.009 |
| **Medical history** | | | |
| CAD, n (%) | 93 (64.6) | 191 (57.9) | 0.171 |
| HT, n (%) | 83 (57.6) | 166 (50.3) | 0.141 |
| DM, n (%) | 46 (31.9) | 114 (34.5) | 0.582 |
| CHF, n (%) | 55 (38.2) | 81 (24.5) | 0.003 |
| Stroke, n (%) | 19 (13.2) | 29 (8.8) | 0.399 |
| CKD, n (%) | 10 (6.9) | 19 (5.8) | 0.332 |
| STEMI, n (%) | 4 (2.9) | 5 (1.6) | 0.367 |
| **Laboratory examination** | | | |
| HDL-C (mmol/L) | 1.06 (0.31 ) | 1.05 (0.28) | 0.717 |
| LDL-C (mmol/L) | 2.79 (0.97) | 2.85 (1.30) | 0.632 |
| HGB (g/L) | 125.4 (20.42) | 134.07 (17.48) | <0.001 |
| PLT (10^9/L) | 232.07 (78.89) | 229.64(65.70) | 0.748 |
| BUN (mmol/L) | 6.61 (3.01) | 6.10 (2.45) | 0.073 |
| Cr (umol/L) | 90.37 (68.83) | 88.57 (68.13) | 0.793 |
| UA (umol/L) | 390.26 (127.46) | 396.42 (114.20) | 0.613 |
| potassium (mmol/L) | 3.99 (0.53) | 4.02 (0.41) | 0.626 |
| sodium (mmol/L) | 140.62 (3.52) | 140.62 (10.52) | 0.996 |
| **ECG** | | | |
| RV5 (mV) | 1.42 [1.08, 2.04] | 1.34 [1.00, 1.70] | 0.011 |
| RV6 (mV) | 1.21 [0.85, 1.61] | 1.09 [0.80, 1.39] | 0.005 |
| RaVL (mV) | 0.47 [0.28, 0.67] | 0.33 [0.17, 0.50] | <0.001 |
| SV1 (mV) | -0.83 [-1.29, -0.50] | -0.73 [-1.01, -0.50] | 0.017 |
| SV3 (mV) | -1.08 [-1.45, -0.61] | -0.88 [-1.24, -0.57] | 0.013 |
| Cornell voltage LVH, n (%) | 16 (11.1) | 3 (1.0) | <0.001 |
| Sokolow-Lyon LVH, n (%) | 25 (17.4) | 4 (1.3) | <0.001 |
| **Echocardiography** | | | |
| LVEF (%) | 61.27 (11.94) | 67.44 (5.35) | <0.001 |
| LVEDD (mm) | 50.38 (6.81) | 43.82 (3.80) | <0.001 |
| LVPW (mm) | 10.33 (1.14) | 9.48 (0.99) | <0.001 |
| LVMI ( g/m^2^ ) | 125.92 (33.00) | 85.99 (15.93) | <0.001 |
| IVS (mm) | 11.59 (1.86) | 10.33 (1.37) | <0.001 |
| **Treatment** | | | |
| ACEI, n (%) | 31 (21.5) | 48 (14.5) | 0.061 |
| ARB, n (%) | 40 (27.8) | 79 (23.9) | 0.375 |
| [spirolactone, n (%)](javascript:;) | 21(14.6) | 37 (11.2) | 0.303 |
| CCB, n (%) | 48 (33.3) | 117 (35.3) | 0.656 |
| BB, n (%) | 89 (61.8) | 207 (62.7) | 0.849 |
| diuretic, n (%) | 31 (21.5) | 65 (19.7) | 0.648 |

Abbreviations are shown as Table 1

**Supplementary Table 4**  Diagnostic performance of different CNN-LSTM models

|  | AUC | sensitivity | specificity | PPV | NPV | F1-score |
| --- | --- | --- | --- | --- | --- | --- |
| Control vs LVH | 0.622 | 68% | 57% | 61% | 64% | 64% |
| Control-M vs LVH-M | 0.659 | 72% | 60% | 64% | 68% | 68% |
| Control-F vs LVH-F | 0.586 | 50% | 71% | 64% | 59% | 56% |
| Control-M vs concentric-M | 0.662 | 62% | 70% | 68% | 65% | 65% |
| Control-M vs eccentric-M | 0.677 | 65% | 71% | 69% | 67% | 67% |
| Control-F vs concentric-F | 0.580 | 48% | 68% | 60% | 57% | 53% |
| Control-F vs eccentric-F | 0.578 | 47% | 69% | 60% | 56% | 53% |

Abbreviations are shown as Supplementary Table 1
